# Increased carotid intima-media thickness in scuba divers

**Published:** 2014

**Authors:** Masoud Mehrpour, Saeed Rezaali, Narges Sadat Shams-Hosseini

**Affiliations:** 1 Department of Neurology, School of Medicine, Iran University of Medical Sciences AND Iranian Center of Neurological Research (ICNR), Tehran University of Medical Sciences, Tehran, Iran; 2 Department of Neurology, School of Medicine, Islamic Azad University, Tehran Medical Branch, Tehran, Iran; 3 Department of Occupational and Environmental Medicine AND Center for Research on Occupational Diseases, Tehran University of Medical Sciences, Tehran, Iran

**Keywords:** Intima-Media Thickness, Scuba Diver, Cerebrovascular Disease

## Abstract

**Background:**

Scuba divers work in high pressure conditions which may cause some changes in physiological status to adapt to this situation. In this study, the carotid intima-media thickness (CIMT) was assessed in divers as a risk factor of cerebrovascular and cardiovascular disorders.

**Methods:**

This historical cohort study was performed on 16 male professional scuba divers as case group and 30 healthy people as controls with age range of 26-66 years. CIMT of both carotids of supine participants was measured by a 10 MHz linear ultrasonic probe quantitatively. Relationship between experience of diving and carotid IMT was evaluated.

**Results:**

All the participants were males (mean age 42.9 ± 10.58. and for the control group was (47.05 ± 12.31 years). The mean right CIMT in divers and control group was 524.31 ± 149.40 and 443.66 ± 59.62 micrometer, respectively. Furthermore, the mean left CIMT in divers and control group was 624.57 ± 116.15 and 458.44 ± 49.56 micrometer, respectively.

**Conclusion:**

The findings demonstrated that long-term occupational diving leads to increased intima-media thickness in scuba divers.

## Introduction

Divers work in high pressure conditions which may cause some changes in their physiological status to adapt to this situation. Some data demonstrated divers at risk of thrombophilic events, cerebrovascular, cardiovascular and respiratory diseases.^1, 2^

In adults, increased carotid intima-media thickness (CIMT) assessed by ultrasonography is a valid predictor of cerebrovascular events.^3, 4^

Hyperbaric condition of divers is thought to be associated with a higher incidence of cerebral ischemic events.^5^ Long-term hyperbaric exposure in divers is considered as an occupational risk factor for brain ischemic events and consequent stroke. According to the literature review, most of the previous studies either have indirectly evaluated this relationship in artificial situation of high altitudes or reported only a limited number of cases of divers. Thus, our study was designed to assess intima-media thickness (IMT) in divers as a risk factor of cerebrovascular events.^6^

## Materials and Methods

### Study population

This historical cohort study was carried out in Firoozgar Hospital affiliated to Iran University of Medical Sciences, Tehran, Iran during June to October 2012. Informed consent was obtained from all the participants, and the study was approved by the Ethics Committee of Iran University of Medical Sciences.

The study groups consisted of 16 professional male scuba divers as case group and 30 healthy people as controls with age range of 26-66 years. All participants had at least a history of two years working without any previous experience of cerebrovascular disease or barotrauma events. Diving depth was 25-50 meters and the divers had different diving records. The eligible participants were selected by random sampling and each diver completed a questionnaire that included demographic data, medical and diving history.

Ultrasound examinations were performed using the 10 MHz linear ultrasonic probe. Antero-oblique insonation, far-wall carotid IMT was visualized bilaterally at 3 sites and the relationship between the experience of diving and carotid IMT was evaluated.

### Statistical analysis

The association between IMT of both carotids and scuba diving was evaluated by Mann-Whitney U-test. We used Spearman's correlation test to find association between quantitative data such as IMT of left and right carotid arteries, age, BMI and experience. Then, comparison of IMT of both carotids and experience of diving for age was performed by linear regression model.

## Results

Totally, 16 divers aged 21-60 years participated in this study. All participants were male with a mean age of 43.41 ± 1.1 years and 27 years (between 2-39 years) median experience of occupational diving. Four participants were smoker (10.5%). One of the divers was retired and had worked as diver for 39 years. Demographic characteristics were similar in both groups. Baseline characteristics of the divers and control group are shown in Table 1.

**Table 1:** Baseline characteristics of the divers and control group

Variables	Divers	Controls	P
Age (Mean ± SD)	42.9 ± 10.58	47.05 ± 12.31	0.26
Experience of diving	27	-	
BMI (Mean ±SD)	27.11 ± 3.14	25.15 ± 4.48	0.13
Marital status			
Married	12 (80%)	16 (93%)	

**Diagram 1:**
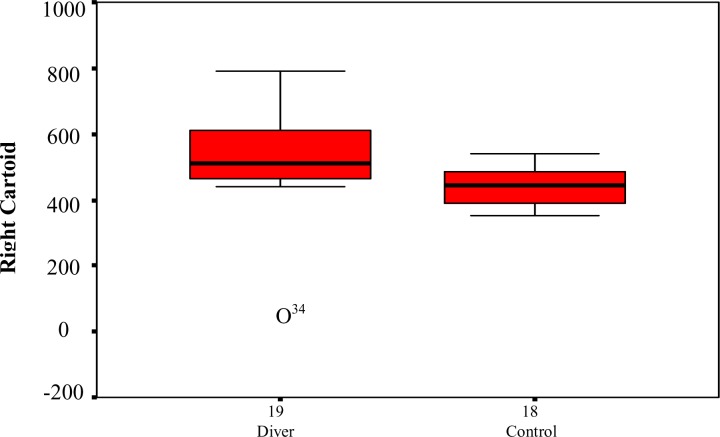
Right carotid intima-media thickness

**Diagram 2:**
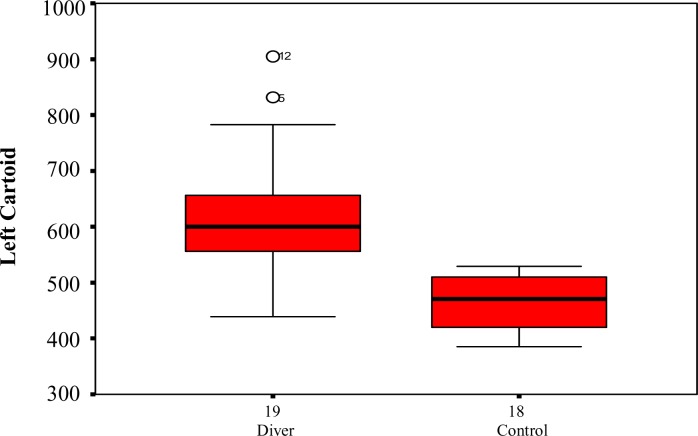
Left carotid intima-media thickness

The mean right CIMT in divers and control group was 524.31 ± 149.40 and 443.66 ± 59.62 micrometer, respectively. Furthermore, the mean left CIMT in divers and control group was 624.57 ± 116.15 and 458.44 ± 49.56 micrometer, respectively. IMT of right and left carotids arteries in scuba divers were higher than non-divers (P = 0.003 and P < 0.001, respectively) (Diagrams 1 and 2).

There was not any statistical significant difference between body mass index (BMI) and age in divers and control group (P > 0.05). Although the left carotid IMT was associated with experience of diving (P = 0.03; r = 0.5), the right one was not associated with it (P = 0.7). Divers with higher experience showed increase left IMT.

## Discussion

We performed this study to assess and compare IMT of carotid in divers as representatives of hyperbaric conditions. While trying to explore these new features of cerebrovascular investigations, some novel findings were expected to be revealed. There is no evidence to IMT of both carotids and scuba diving. Our findings showed that IMT of both carotids in scuba divers were increased than non-divers and were associated with experience of diving and BMI in scuba divers.

Study of Boussuges et al. demonstrated numerous hemodynamic changes one hour after an open-sea scuba diving.^7^

A previous study by Moen et al. was performed on Norwegian professional divers. They found widespread diffusion and perfusion abnormalities in different parts of brain hemodynamic of divers compared with controls.^8^

Candito et al. detected that divers are susceptible to thrombophilic events.^2^ Dujic demonstrated that breath holding can cause cerebrovascular, cardiovascular and respiratory diseases^1^. These findings supported the hypothesis that diving might be at a risk of cerebrovascular disease.

Our finding showed that IMT of the left carotid arteries was significantly associated with diving. Some evidences showed CIMT elevation in middle aged and elderly patients with lung function impairment even without significant respiratory disease.^9^

We had some limitations in this study. One such limitation was small sample size. It is worth mentioning to know that our findings can explain some of the most common clinical symptoms of professional divers. We could not illustrate relevant cause of increased IMT, but we suggest that chronic exposure to hyperbaric condition might cause increase in trombophilic events and respiratory disease that result in increased left IMT in divers. We strongly recommend further evaluation of IMT in divers by performing longitudinal evaluations.
